# Frailty Component Trajectories After Restoration of Kidney Function Among Kidney Transplant Recipients

**DOI:** 10.1093/geroni/igaa057.864

**Published:** 2020-12-16

**Authors:** Nadia Chu, Xiaomeng Chen, Dorry Segev, Mara McAdams-DeMarco

**Affiliations:** 1 Johns Hopkins School of Medicine, Baltimore, Maryland, United States; 2 Johns Hopkins Bloomberg School of Public Health, Baltimore, Maryland, United States

## Abstract

Frailty is associated with decreased access to kidney transplantation (KT) and poor post-KT outcomes. Little is known about how an acute stressor, like KT, can impact the five physical frailty phenotype (PFP) criteria. We conducted a two-center prospective cohort study (2009-2019) of adult patients undergoing KT. PFP criteria were measured at KT admission, 1 month, 3 months, 6 months, 1 year, and annually thereafter post-KT. We used adjusted mixed effects models with fixed and random effects for person and time to describe repeated measures of continuous criteria components (weight, gait speed, grip strength, activity). We used an adjusted generalized estimating equation to quantify longitudinal, binomial response patterns of exhaustion. Among 1,410 KT recipients (mean age=53) followed for a mean of 1.9 years (IQR=0.1-3.2), 46.8% had low activity, 46.7% weakness, 29.0% exhaustion, 16.1% slowness, and 14.3% unintentional weight loss at KT admission. Among continuous components, weight worsened (0.3lb/month, 95%CI:0.2,0.4), while grip strength (0.09kg/month, 95%CI: 0.07,0.11) and activity (5.8Kcal/month, 95%CI: 3.3, 8.2) improved post-KT; gait speed remained stable (-0.0004s/month, 95%CI: -0.01, 0.0005). Additionally, likelihood of transitioning from being exhausted did not change (OR=1.0, 95%CI: -0.01, 0.0005). Trajectories differed by age, such that improvements were observed among younger recipients (<65 years), but not among older recipients (≥65 years) (p-interactions<0.05). After undergoing a common surgical stressor, KT recipients demonstrated weight gain as well as improvements in strength and activity. Despite benefits of restoration of kidney function, clinicians should consider monitoring KT recipients for persistent weight gain, exhaustion, and slowness post-KT, particularly among older adults.

